# Development and Validation of 3‐Year Atrial Fibrillation Prediction Models Using Electronic Health Record With or Without Standardized Electrocardiogram Diagnosis and a Performance Comparison Among Models

**DOI:** 10.1161/JAHA.121.024045

**Published:** 2022-06-14

**Authors:** Yunjin Yum, Seung Yong Shin, Hakje Yoo, Yong Hyun Kim, Eung Ju Kim, Gregory Y. H. Lip, Hyung Joon Joo

**Affiliations:** ^1^ Department of Biostatistics Korea University College of Medicine Seoul Republic of Korea; ^2^ Cardiovascular and Arrhythmia Center Chung‐Ang University Hospital Seoul Republic of Korea; ^3^ Korea University Research Institute for Medical Bigdata Science Korea University Seoul Republic of Korea; ^4^ Department of Internal Medicine Korea University Ansan Hospital Seoul Republic of Korea; ^5^ Cardiovascular Center Korea University Guro Hospital Seoul Republic of Korea; ^6^ Liverpool Center for Cardiovascular Science University of Liverpool Liverpool UK; ^7^ Aalborg University Aalborg Denmark; ^8^ Department of Medical Informatics Korea University College of Medicine Seoul Republic of Korea; ^9^ Cardiovascular Center Korea University Anam Hospital Seoul Republic of Korea

**Keywords:** atrial fibrillation, ECG, electronic health record, risk prediction, Atrial Fibrillation, Electrophysiology

## Abstract

**Background:**

Improved prediction of atrial fibrillation (AF) may allow for earlier interventions for stroke prevention, as well as mortality and morbidity from other AF‐related complications. We developed a clinically feasible and accurate AF prediction model using electronic health records and computerized ECG interpretation.

**Methods and Results:**

A total of 671 318 patients were screened from 3 tertiary hospitals. After careful exclusion of cases with missing values and a prior AF diagnosis, AF prediction models were developed from the derivation cohort of 25 584 patients without AF at baseline. In the internal/external validation cohort of 117 523 patients, the model using 6 clinical features and 5 ECG diagnoses showed the highest performance for 3‐year new‐onset AF prediction (C‐statistic, 0.796 [95% CI, 0.785–0.806]). A more simplified model using age, sex, and 5 ECG diagnoses (atrioventricular block, fusion beats, marked sinus arrhythmia, supraventricular premature complex, and wide QRS complex) had comparable predictive power (C‐statistic, 0.777 [95% CI, 0.766–0.788]). The simplified model showed a similar or better predictive performance than the previous models.

In the subgroup analysis, the models performed relatively better in patients without risk factors. Specifically, the predictive power was lower in patients with heart failure or decreased renal function.

**Conclusions:**

Although the 3‐year AF prediction model using both clinical and ECG variables showed the highest performance, the simplified model using age, sex, and 5 ECG diagnoses also had a comparable prediction power with broad applicability for incident AF.

**Nonstandard Abbreviations and Acronyms**
CHARGE‐AF: Cohorts for Heart and Aging Research in Genomic Epidemiology Model for Atrial Fibrillation
OMOP‐CDM: observational medical outcomes partnership–common data model
SNOMED CT: systematized nomenclature of medicine–clinical terms

**Clinical Perspective**

**What Is New?**

- For improved atrial fibrillation prediction, ECG, biomarkers, and clinical risk factors with balanced simplicity and applicability are important, their possible combinations are induced, and their performance was compared.
- With age, sex, and 5 automated ECG diagnoses, a simplified 3‐year atrial fibrillation prediction model showed a comparable performance, especially in patients without closely associated comorbidities, such as heart failure and stroke.

**What Are the Clinical Implications?**

- According to a given clinical situation, either simplified model or full model can predict 3‐year atrial fibrillation with improved performance.

Ischemic stroke is a major health care problem globally, leading to death, disability, and an impaired quality of life. Among the risk factors for ischemic stroke, atrial fibrillation (AF) is a modifiable risk factor that can effectively reduce the risk of stroke through appropriate anticoagulation therapy.^1^ However, early diagnosis of AF is practically difficult because there are few characteristic symptoms in the early stages of AF, and there are no clinical symptoms despite a similarly poor outcome in asymptomatic and symptomatic patients.^2^, ^3^, ^4^ A previous meta‐analysis demonstrated that the overall AF detection rate was 11.5% after an ischemic stroke or a transient ischemic attack.^5^ If AF can be diagnosed or predicted earlier, the incidence and serious consequences of strokes can be substantially reduced by appropriate AF management.^6^

Clinical risk factors that are important in AF development have been introduced. Several novel biomarkers have also been reported to show comparable accuracy for predicting AF.^7^ However, these biomarkers are not yet widely applied in real‐world practice because of their limited availability and cost. The pathophysiological characteristics of AF include complex and heterogeneous mechanisms, which makes it difficult to develop simple and clinically available AF prediction estimates that can be easily applied in real‐world practice with sufficient accuracy.

The introduction of electronic health records (EHRs) has made it easier to establish clinical big data, and studies using this have been actively conducted. Hulme et al proposed an AF prediction model using EHR data.^8^ They developed their EHR‐AF risk score based on 16 selected variables; its C‐statistic was 0.76. External validation study, including the other AF prediction models, showed 0.80 of the C‐index from EHR‐AF, 0.80 from Cohorts for Heart and Aging Research in Genomic Epidemiology Model for Atrial Fibrillation (CHARGE‐AF), 0.68 from C2HEST models (coronary artery disease or chronic obstructive pulmonary disease [1 point each]; hypertension [1 point]; elderly [aged ≥75 years; 2 points]; systolic heart failure [2 points]; and thyroid disease [hyperthyroidism; 1 point]), and 0.72 from CHA_2_DS_2_‐VASc (congestive heart failure, hypertension, age of ≥75 years, diabetes, stroke or transient ischemic attack, vascular disease, age of 65 to 74 years, and sex category).^9^ These models predict the occurrence of AF using only simple information that can be obtained through questionnaires or physical measurements, such as disease history, smoking status, and blood pressure. Considering that EHR includes the results of various test equipment in hospitals, the predictive power of AF occurrence can be expected to improve by using this information.

The 12‐lead ECG is a basic test used to evaluate the electrophysiological state of the heart. Modern ECG machines provide computerized ECG diagnoses comparable to the physicians' interpretations.^10^, ^11^ Automated ECG interpretation is cost‐ and time‐effective, with minimized intraobserver and interobserver variability. The present study aimed to apply computerized standard ECG diagnosis to the development of an AF prediction model and to validate its performance.

## Methods

All data and supporting materials have been provided with the published article. Study patients were identified from the EHRs of the 3 tertiary hospitals (Korea University Anam Hospital for model derivation and internal validation, n=397 905; and Korea University Guro/Ansan Hospital for external validation, n=133 813/139 600, respectively). EHR data from a single hospital were used for the development and internal validation of the AF prediction algorithm. External validation was performed using data from 2 other hospitals located in different districts and cities. The study protocol was approved by the institutional review boards of each institute. Written informed consent was waived because of the retrospective study design of anonymized data, with minimal risk to the patients. The study complied with the principles of the Declaration of Helsinki.

**Figure 1:**
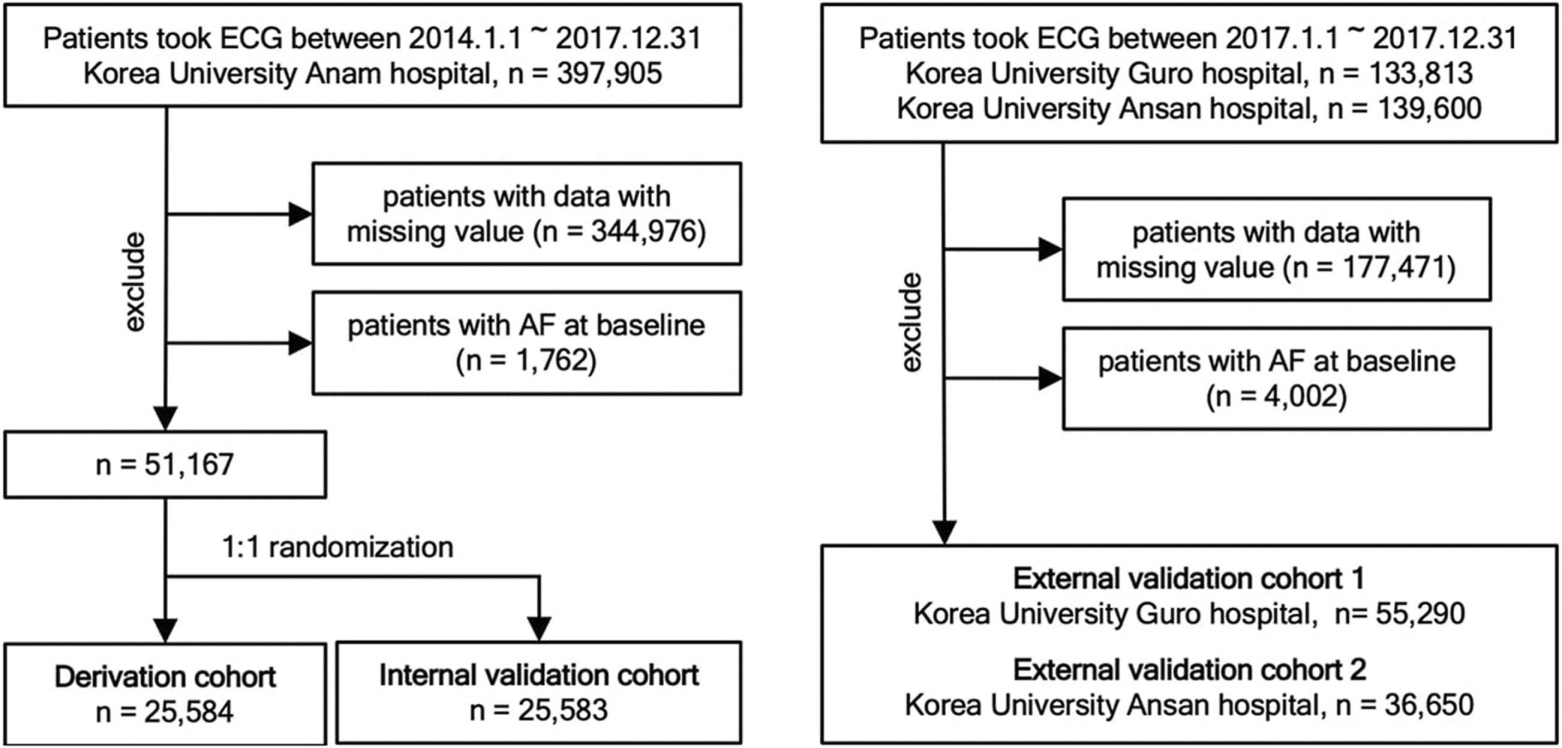
Study design. AF indicates atrial fibrillation.

For the development and internal validation, 397 905 patients who underwent ECG recordings between January 1, 2014, and December 31, 2017, were screened (Figure 1). Baseline covariates were ascertained 1 year before the follow‐up start date (index date), and covariates included in the analysis were measurements closest to the follow‐up start date. A total of 344 976 patients with missing covariate values, including vital signs and laboratory data, were excluded. Of these, 1762 patients with AF confirmed through ECG or the diagnosis code on the EHR before the index visit were also excluded. Finally, 51 167 patients were randomly divided into the derivation set (n=25 584) and internal validation set (n=25 583).

For the external validation, data of 55 290 and 36 650 patients were extracted from the patients who underwent ECG recordings at the Guro Hospital and Ansan Hospital, respectively, from September 1, 2017, to December 31, 2017, and from January 1, 2017, to December 30, 2017, respectively.

### Ascertainment of Clinical Characteristics

The clinical characteristics of patients were extracted from the diagnosis code, clinical diagnosis name, medication and prescription history, outpatient charts, hospital discharge records, and examination records in the EHR. The detection of AF during the follow‐up period was based on diagnosis codes and ECG reports. The validated algorithm for AF detection was adopted from a previous study.^12^ AF was detected when (1) AF was documented on ECG, AF/flutter ablation, or cardioversion, and (2) ≥2 hospital visits were recorded with the *International Classification of Diseases, Ninth Revision* (*ICD‐9*), diagnosis codes for AF until censored by death or the last follow‐up. The positive predictive value of AF was 92%.

Potential clinical risk factors were selected on the basis of previous studies.^8^, ^13^, ^14^ These included age, sex, smoking status, alcohol consumption, height, body weight, systolic and diastolic blood pressure, underlying comorbidities (hypertension, diabetes, dyslipidemia, chronic kidney disease [CKD], thyroid disease, heart failure, valvular heart disease, coronary artery disease, stroke, peripheral arterial disease, and chronic obstructive pulmonary disease), medications (antihypertensive medication and insulin), and laboratory findings (glucose, hemoglobin A1c, total cholesterol, low‐density lipoprotein cholesterol, high‐density lipoprotein cholesterol, and creatinine). The values of these variables were taken from 1 year before the index date to the closest value to the index date.

Clinical risk factors were defined by combining diagnostic codes (the *International Classification of Diseases, Tenth Revision* [*ICD‐10*], codes) and laboratory test results. Hypertension was defined when patients were diagnosed with I10 to I15 of the *ICD‐10* codes or treated with antihypertensive drugs. Diabetes was defined when patients had been diagnosed with E10 to E14 of the *ICD‐10* codes or treated with oral hypoglycemic drugs or insulin or whose fasting plasma glucose level was ≥126 mg/dL or hemoglobin A1c was ≥6.5%. Patients with dyslipidemia were defined as those diagnosed with E78.0.6, E78.8, E78.9, E88.8, or E88.9 of the *ICD‐10* codes or treated with any lipid‐lowering agents or whose total cholesterol level ≥240 mg/dL, low‐density lipoprotein level ≥160 mg/dL, high‐density lipoprotein level <40 mg/dL, or triglyceride level ≥200 mg/dL. Patients who had been diagnosed with N18.0 to N18.5 and N18.8 to N18.9 were defined as having CKD.

Other comorbidities were defined on the basis of the *ICD‐10* codes. These included heart failure (I50.0, I50.1, and I50.9), mitral valvular stenosis (I05.0 and I05.2), other valvular heart disease (I05.1–I05.2, I05.8–I05.9, I06.0–I06.2, I06.8–I06.9, I07.0–I07.1, I07.8–I07.9, I08.0–I08.1, I08.3, I34.1–I34.2, I34.8–I34.9, I35.0–I35.2, I35.8–I35.9, I36.1, I36.8, I37.0–I37.2, I38, I39.0–I39.2, and I39.8), thyroid disease (E02–E03, E05–E07, E35, E89.0, H06.2, O90.5, P72.2, and R94.6), chronic obstructive pulmonary disease (J44.0 and J44.8–J44.9), coronary artery disease (I20.0, I21, I22, and I25.2), atherosclerotic ischemic stroke/transient ischemic attack (I63, I64, and G45), and peripheral vascular disease (I70.0, I70.1, I70.2, I70.8, and I70.9).

### Standardization of Computerized ECG Diagnosis

The hospitals participating in this study used 12‐lead ECG machines from 3 vendors (GE Medical System, Philips Medical Systems, and Nihon Kohden). ECG machines automatically generated ECG diagnoses and ancillary descriptions using the approved computerized algorithm of each vendor.

For example, atrioventricular block was defined in cases with variable atrioventricular blocks, including second‐degree atrioventricular block, complete heart block, or atrioventricular dissociation. Fusion beats were ectopically shaped QRS complexes with 100 ms of the expected RR interval. Marked sinus arrhythmia was defined as a range of RR intervals exceeding 40% of the average RR interval. The supraventricular premature complex was a premature, normally shaped QRS complex without the preceding P waves. A wide QRS complex indicates a wide QRS rhythm (QRS duration >120 ms and ventricular rate between 40 and 120 beats per minute). The ECG findings of the ECG diagnosis and ancillary descriptions were in the free‐text format and placed in the “statement” section in the original XML files. These free texts were transformed into the terminology of the systematized nomenclature of medicine–clinical terms (SNOMED CT) and its cross‐referenced terminology of the observational medical outcomes partnership–common data model (OMOP‐CDM).

SNOMED CT is a systematic, international, standardized medical terminology system that is used to effectively record and communicate clinical data in EHRs to improve patient care in major countries, such as the United States, Canada, and the United Kingdom.^15^ OMOP provides a common data schema with its own coding system, adopting international standard terminologies such as SNOMED CT. Because the CDM includes various international standard terminologies, it could enhance international big data analysis research through multicenter distributed network research and various clinical studies.^16^

Practically, standard terms and code mapping for ECG reports were performed using a web‐based software, which is an integrated algorithm using cosine similarity and rule‐based hierarchy (available at cdal.korea.ac.kr/ECG2CDM). This software is optimized for mapping ECG reports from the 3 vendors (GE, Philips, and Nihon Kohden) into standard terms and codes of the OMOP‐CDM. The overall accuracy of the software was >99% for all 3 ECG machine vendors. OMOP‐CDM terms and codes can also be easily converted to SNOMED CT codes and terms using the CONCEPT table at http://athena.ohdsi.org. For example, the OMOP‐CDM concept name “ECG normal” (concept identifier: 4065279) originated from the SNOMED CT name “electrocardiogram normal (finding)” (SNOMED code 164854000). Both the OMOP‐CDM concept identifier “4065279” and SNOMED code 164854000 define “normal ECG.” Finally, the ECG database of the present study included 130 ECG diagnoses.

### Statistical Analysis

A Cox proportional hazards regression model was used to develop the 3‐year AF prediction model. Clinical risk factors for multivariable analysis were selected when the *P* value of the univariate analysis was <0.1. ECG diagnoses were selected when their prevalence was >0.02%, and the *P* value of the univariate analysis was <0.1. With the selected ECG features and 10 clinical features (age, sex, hypertension, diabetes, dyslipidemia, heart failure, valvular heart disease, coronary artery disease, stroke, and CKD), multivariable Cox proportional hazards regression models were fitted with a backward elimination approach, retaining variables that satisfied a significance level of 0.05. Several multivariable Cox proportional hazards regression models were developed. C‐statistic and net reclassification index analysis was used to estimate performance of each model.

The models were compared with previously published models. These included the following: (1) CHARGE‐AF included 11 variables (age [5 years], race [White], height [10 cm], weight [15 kg], systolic blood pressure [20 mm Hg], diastolic blood pressure [10 mm Hg], current smoking, antihypertensive medication use, diabetes, heart failure, and myocardial infarction)^13^; (2) C2HEST included 12 variables (old age [75 years], sex, hypertension, diabetes, dyslipidemia, CKD, chronic obstructive pulmonary disease, thyroid disease, coronary artery disease, vascular disease, heart failure, and valvular heart disease)^14^; and (3) EHR‐AF included 16 variables (age, sex, race, smoking, height, weight, hypertension, diastolic blood pressure, dyslipidemia, CKD, thyroid disease, coronary artery disease, vascular disease, transient ischemic attack, heart failure, and valvular heart disease).^8^ Discrimination of the models was evaluated using C‐statistic for time‐to‐event data. Net reclassification index analysis was used to estimate performance of each model.

The cumulative new‐onset AF incidence was demonstrated using Kaplan‐Meier survival curves with a log‐rank test. In this analysis, patients in the validation cohorts were divided into 3 groups of 3‐year new‐onset AF risks (<1%, 1%–3%, and >3%), which were calculated using the models developed in this study.

Comparisons between the groups were performed using the independent Student *t*‐test or Mann‐Whitney test for continuous variables and the χ^2^ test or Fisher exact test for categorical variables. All tests were 2 tailed, and *P*<0.05 was considered statistically significant. All statistical analyses were performed using SAS version 9.4 (SAS Institute) and R version 3.6.1, with the rms, survminer, and survival packages.

## Results

### Baseline Characteristics of the Derivation, Internal Validation, and External Validation Cohorts

The baseline characteristics of the patients are presented in Table 1. Despite the different baseline characteristics of the external validation cohort, caused by different data sources, there were no significant differences in terms of AF incidence (1.1% in the internal validation cohort versus 1.2% in the external validation cohorts 1 and 2).

**Table 1:** Baseline Clinical and ECG Characteristics of the Derivation, Internal Validation, and External Validation Cohorts

Variable	Derivation and internal validation cohorts (n=51 167)	External validation cohort 1 (n=55 290)	External validation cohort 2 (n=36 650)	*P* value
Clinical characteristics				
Age, y	50.56±21.77	57.16±17.61	52.84±18.93	<0.001
Men	25 910 (50.64)	26 956 (48.75)	18 903 (51.58)	<0.001
Current smoker	8284 (16.19)	4263 (7.71)	3404 (9.29)	<0.001
Alcohol drinking	11 124 (21.74)	5666 (10.25)	4141 (11.3)	<0.001
Systolic BP, mm Hg	119.66±15.38	123.64±16.52	124.77±17.56	<0.001
Diastolic BP, mm Hg	74.46±10.69	75.71±11.86	75.85±12.43	<0.001
Hypertension	12 859 (25.13)	23 181 (41.93)	11 569 (31.57)	<0.001
Diabetes	17 489 (34.18)	19 878 (35.95)	12 375 (33.77)	<0.001
Dyslipidemia	13 324 (26.04)	23 369 (42.27)	12 548 (34.24)	<0.001
Chronic kidney disease	942 (1.84)	2024 (3.66)	1345 (3.67)	<0.001
Thyroid disease	1035 (2.02)	2334 (4.22)	2933 (8)	<0.001
COPD	48 (0.09)	1051 (1.9)	237 (0.65)	<0.001
Heart failure	417 (0.81)	615 (1.11)	568 (1.55)	<0.001
VHD				
MV stenosis	22 (0.04)	40 (0.07)	28 (0.08)	<0.001
Other VHD	128 (0.25)	226 (0.41)	83 (0.23)	
Coronary artery disease	3157 (6.17)	7449 (13.47)	3339 (9.11)	<0.001
Stroke	2423 (4.74)	3357 (6.07)	1665 (4.54)	<0.001
Peripheral arterial disease	194 (0.38)	759 (1.37)	598 (1.63)	<0.001
ECG diagnosis (top 20)				
Normal sinus rhythm	19 537 (38.18)	25 129 (45.45)	7779 (21.23)	<0.001
Sinus rhythm (bradycardia)	3676 (7.18)	5717 (10.34)	1356 (3.7)	<0.001
T wave (abnormal)	2478 (4.84)	3498 (6.33)	1413 (3.86)	<0.001
Sinus rhythm	2430 (4.75)	3000 (5.43)	3698 (10.09)	<0.001
LVH	1288 (2.52)	1955 (3.54)	938 (2.56)	<0.001
Sinus rhythm (tachycardia)	1174 (2.29)	1649 (2.98)	797 (2.17)	<0.001
QT interval (prolonged)	1048 (2.05)	1305 (2.36)	719 (1.96)	<0.001
Sinus arrhythmia	944 (1.84)	746 (1.35)	431 (1.18)	<0.001
Left‐axis deviation	901 (1.76)	1359 (2.46)	535 (1.46)	<0.001
Right‐axis deviation	869 (1.7)	912 (1.65)	441 (1.2)	<0.001
Atrioventricular block (first degree)	838 (1.64)	1535 (2.78)	480 (1.31)	<0.001
Atrioventricular block	825 (1.61)	1557 (2.82)	291 (0.79)	<0.001
Myocardial infarction (inferior)	799 (1.56)	1249 (2.26)	383 (1.05)	<0.001
Myocardial ischemia (lateral)	762 (1.49)	1067 (1.93)	387 (1.06)	<0.001
ST‐T abnormality (nonspecific)	652 (1.27)	1205 (2.18)	287 (0.78)	<0.001
RBBB	636 (1.24)	978 (1.77)	278 (0.76)	<0.001
Myocardial ischemia (anterior)	607 (1.19)	750 (1.36)	301 (0.82)	<0.001
Early repolarization	477 (0.93)	690 (1.25)	522 (1.42)	<0.001
Ventricular premature complex	442 (0.86)	927 (1.68)	225 (0.61)	<0.001
ST‐segment elevation	334 (0.65)	529 (0.96)	464 (1.27)	<0.001

Values are presented as number (percentage) or mean±SD. BP indicates blood pressure; COPD, chronic obstructive pulmonary disease; LVH, left ventricular hypertrophy; MV, mitral valve; RBBB, right bundle‐branch block; and VHD, valvular heart disease.

### Development of 3‐Year New‐Onset AF Prediction Models

Clinical risk factors and ECG diagnoses were screened in the derivation cohort using univariate Cox regression analysis (Table S1). Four types of multivariable AF prediction models were developed (Table 2).

**Table 2:** Multivariable Cox Regression Models for 3‐Year New‐Onset AF Prediction in the Derivation Cohort

Model	Predictors	HR (95% CI)	*P* value
Model 1 (ECG diagnosis model)	Atrioventricular block	3.07 (1.84–5.14)	<0.001
	Fusion beats	11.72 (4.54–30.30)	<0.001
	Sinus arrhythmia (marked)	6.22 (2.84–13.63)	<0.001
	Supraventricular premature complex	8.99 (4.17–19.38)	<0.001
	Wide QRS complex	4.72 (2.25–9.91)	<0.001
Model 2 (simplified model with ECG diagnosis)	Age	1.06 (1.05–1.07)	<0.001
	Sex	1.55 (1.22–1.98)	<0.001
	Atrioventricular block	1.86 (1.12–3.07)	0.02
	Fusion beats	7.95 (3.11–20.34)	<0.001
	Sinus arrhythmia (marked)	4.56 (2.08–9.99)	<0.001
	Supraventricular premature complex	5.72 (2.70–12.12)	<0.001
	Wide QRS complex	3.68 (1.79–7.58)	<0.001
Model 3 (full model with ECG diagnosis)	Age	1.05 (1.04–1.06)	<0.001
	Sex	1.58 (1.24–2.02)	<0.001
	Chronic kidney disease	1.47 (0.88–2.47)	0.15
	Heart failure	4.09 (2.49–6.72)	<0.001
	VHD		
	MV stenosis	8.44 (2.07–34.46)	0.003
	Other VHD	2.04 (0.79–5.27)	0.14
	Previous stroke	2.59 (1.87–3.58)	<0.001
	Atrioventricular block	1.65 (0.98–2.76)	0.06
	Fusion beats	9.30 (3.64–23.74)	<0.001
	Sinus arrhythmia (marked)	4.21 (1.90–9.31)	<0.001
	Supraventricular premature complex	5.27 (2.43–11.46)	<0.001
	Wide QRS complex	3.26 (1.56–6.83)	0.002
Model 4 (full model without ECG diagnosis)	Age	1.05 (1.05–1.06)	<0.001
	Sex	1.63 (1.27–2.08)	<0.001
	Chronic kidney disease	1.71 (1.03–2.85)	0.04
	Heart failure	4.66 (2.87–7.58)	<0.001
	VHD		
	MV stenosis	7.50 (1.83–30.65)	0.005
	Other VHD	2.10 (0.83–5.31)	0.12
	Previous stroke	2.49 (1.80–3.44)	<0.001

AF indicates atrial fibrillation; HR, hazard ratio; MV, mitral valve; and VHD, valvular heart disease.

Model 1 (ECG diagnosis model) only considered ECG diagnosis as a potential risk factor. Model 2 (simplified model with ECG diagnosis) included age and sex as essential demographic risk factors in addition to model 1. Model 3 (full model with ECG diagnosis) included all potential clinical risk factors and ECG diagnoses. Model 4 (full model without ECG diagnosis) only considered conventional clinical risk factors for model development. Finally, model 1 proposed 5 ECG diagnoses as predictors of the prediction model. Model 3 included 6 clinical risk factors for 5 ECG diagnoses. Model 4 proposed only 6 clinical risk factors without an ECG diagnosis.

The 3‐year AF prediction formula could be derived from the survival function of the Cox regression model. The following equation estimates the predicted AF risk:

(1) $$1-{\left[S_{0}\left(t\right)\right]}^{\exp\left(\sum_{i=1}^{k}\beta_{i}X_{i}-\sum_{i=1}^{k}\beta_{i}\underline{X_{i}}\;\right)},$$

where $S_{0}\left(t\right)$ denotes baseline survival rate at time *t*, $\beta_{i}$ regression coefficient for each predictor, $X_{i}$ denotes values for each predictor, $\underset{\_}{X_{i}}$ denotes mean values for each predictor, and *k* denotes the number of risk factors. Hence, using the coefficient from the model, we computed the probability of developing AF within 3 years as below. For example, the 3‐year AF prediction formula for model 2 (simplified model with ECG diagnosis) is:

(2) $$1-0.996^{\exp\left(0.055X_{\mathrm{age}}+0.440X_{\mathrm{sex}}+0.619X_{\mathrm{AV}\;\text{block}}+2.073X_{\text{Fusion beats}}+1.517X_{\text{Sinus arrhythmia}\;\left(\text{marked}\right)}+1.745X_{\mathrm{SPC}}+1.303X_{\text{Wide}\;\mathrm{QRS}\;\text{complex}}-3.022\right)}$$

### Validation of 3‐Year New‐Onset AF Prediction Models

**Figure 2:**
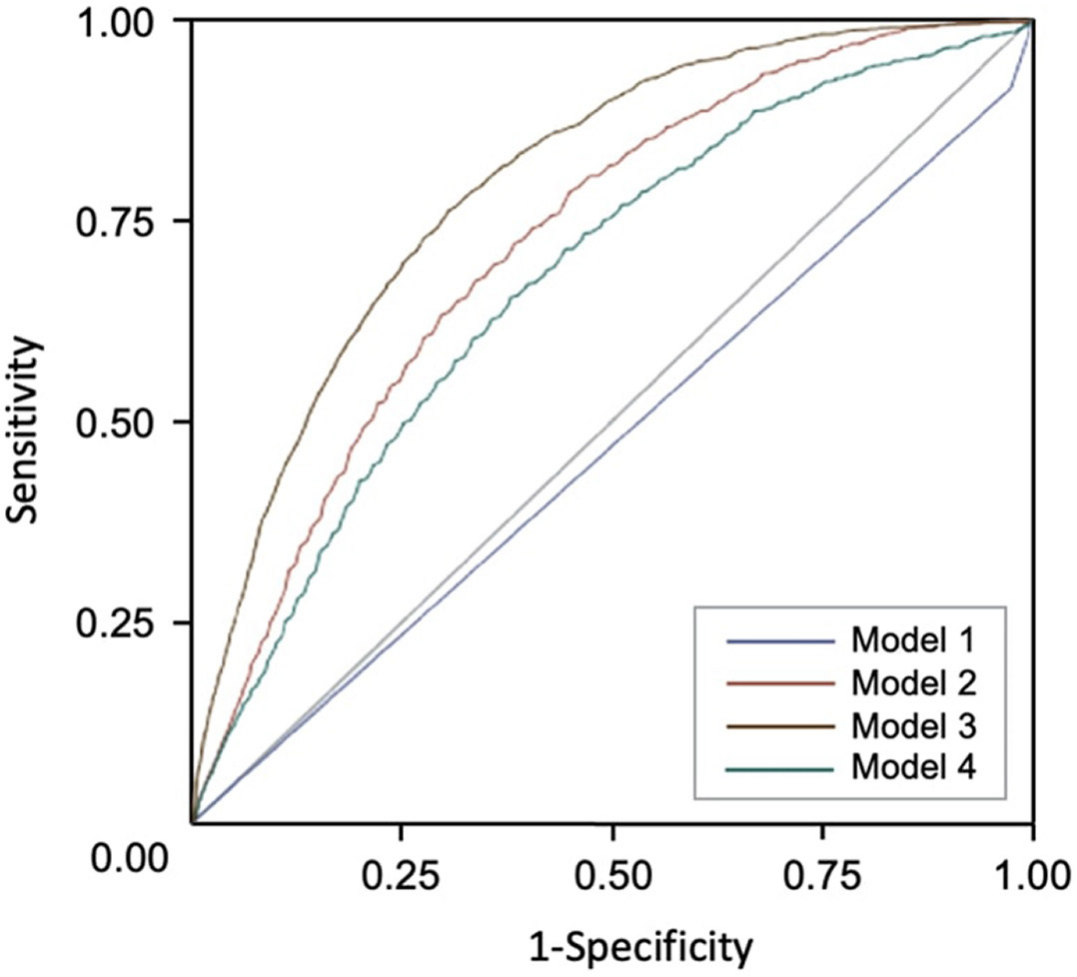
Receiver operating characteristic curves of the proposed models for atrial fibrillation incidence prediction in the total validation cohort population (n=117 523).

Model 1 (ECG diagnosis model) showed the lowest performance, and model 3 (full model with ECG diagnosis) showed the highest performance, in the derivation cohort (Table 3 and Figure 2). This suggests that ECG diagnosis alone is insufficient to predict AF incidence. Model 2 (simplified model with ECG diagnosis) (C‐statistic, 0.777 [95% CI, 0.766–0.788]) showed results comparable to those of model 3 (C‐statistic, 0.796 [95% CI, 0.785–0.806]). Model 4 (full model without ECG diagnosis) (C‐statistic, 0.793 [95% CI, 0.783–0.804]) also showed results comparable to those of model 3. The proposed models showed a similar prediction performance in the internal and 2 external validation cohorts compared with the derivation cohort. In addition, model 2 and model 3 showed higher net reclassification index values compared with model 1 (Table 4). More important, model 2 showed comparable reclassification of AF prediction to model 3. Only 2.4% of patients were better reclassified when the other clinical information was included in model 2.

**Table 3:** C‐Statistic Comparison of 3‐Year New‐Onset AF Prediction Models

Cohort	Model 1 (ECG diagnosis model)	Model 2 (simplified model with ECG diagnosis)	Model 3 (full model with ECG diagnosis)	Model 4 (full model without ECG diagnosis)
Derivation cohort (n=25 584)	0.560 (0.538–0.582)	0.785 (0.761–0.809)	0.807 (0.783–0.831)	0.799 (0.775–0.823)
Internal validation cohort (n=25 583)	0.523 (0.508–0.538)	0.786 (0.763–0.808)	0.800 (0.779–0.822)	0.798 (0.776–0.820)
External validation cohort 1 (n=55 290)	0.541 (0.529–0.553)	0.763 (0.747–0.779)	0.778 (0.762–0.794)	0.775 (0.759–0.791)
External validation cohort 2 (n=36 650)	0.521 (0.510–0.531)	0.795 (0.776–0.813)	0.819 (0.801–0.836)	0.817 (0.799–0.835)
Total validation cohorts (n=117 523)	0.531 (0.523–0.538)	0.777 (0.766–0.788)	0.796 (0.785–0.806)	0.793 (0.783–0.804)

Data in parentheses are 95% CIs. AF indicates atrial fibrillation.

**Table 4:** Reclassification Analysis Using NRI

Pairs	NRI (95% CI)	*P* value
Model 1 vs model 2	0.303 (0.276 to 0.331)	<0.01
Model 1 vs model 3	0.327 (0.3000 to 0.355)	<0.01
Model 1 vs model 4	0.323 (0.294 to 0.352)	<0.01
Model 2 vs model 3	0.024 (0.005 to 0.044)	0.02
Model 4 vs model 2	−0.020 (−0.040 to 0.000)	0.05
Model 4 vs model 3	0.004 (−0.004 to 0.014)	0.34

NRI indicates net reclassification index.

**Figure 3:**
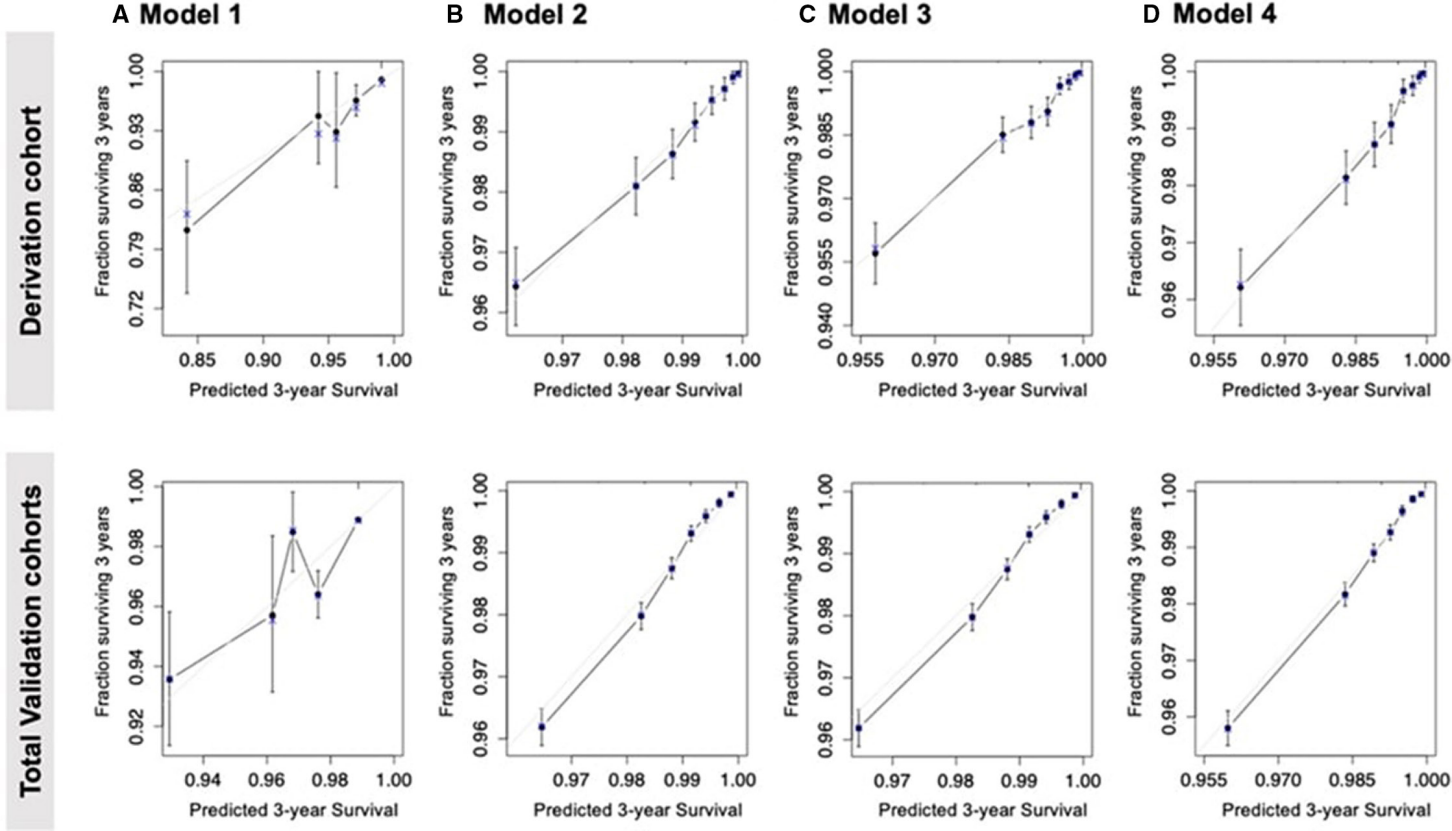
Calibration plot of the proposed models for the derivation and total validation cohorts.

The receiver operating characteristic curve showed that the area under the curve (AUC) of model 3 was the highest at 0.8 (Figure 2). The next AUC ranking was model 2 (AUC, 0.73), model 4 (AUC, 0.68), and model 1 (AUC, 0.47). The AUC values for each model were statistically significant. The calibration plots are shown in Figure 3. The calibration of the proposed models for AF incidence prediction was good as the black line mostly coincided with the gray line, indicating perfect calibration.

### Robustness of the 3‐Year New‐Onset AF Prediction Models

To evaluate the robustness of the proposed models, a subgroup analysis was performed for model 2 (simplified model with ECG diagnosis), model 3 (full model with ECG diagnosis), and model 4 (full model without ECG diagnosis) in the validation cohort (Table 5). Overall, the models developed in this study showed better predictive power in young, female patients and in the group without AF risk factors, such as hypertension and diabetes. The group with the highest C‐statistic was the group without hypertension. The group with the lowest C‐statistic among the clinical risk factor group was the patient group with heart failure in model 2, and the group with an estimated glomerular filtration rate of <30 mL/min per 1.73 m^2^ in models 3 and 4. These results suggest that separate AF prediction models for patients with CKD and heart failure may need to be developed.

**Table 5:** Subgroup Analysis of the 3‐Year New‐Onset AF Prediction Models in Total Validation Cohort Population

Subgroup		No. (%)	AF incidence, %	C‐statistic (95% CI)		
				Model 2	Model 3	Model 4
Age, y	<65	79 721 (67.8)	0.51	0.609 (0.585–0.633)	0.676 (0.651–0.701)	0.664 (0.639–0.689)
	≥65	37 802 (32.2)	2.62	0.565 (0.549–0.581)	0.63 (0.612–0.648)	0.619 (0.601–0.637)
Sex	Men	58 838 (50.1)	1.44	0.762 (0.748–0.776)	0.777 (0.763–0.791)	0.773 (0.759–0.787)
	Women	58 685 (49.9)	0.94	0.784 (0.766–0.802)	0.816 (0.800–0.832)	0.814 (0.798–0.830)
Hypertension	Yes	41 178 (35)	2.10	0.691 (0.673–0.709)	0.725 (0.709–0.741)	0.722 (0.704–0.740)
	No	76 345 (65)	0.70	0.810 (0.794–0.826)	0.823 (0.807–0.839)	0.818 (0.802–0.834)
Diabetes	Yes	41 065 (34.9)	1.85	0.718 (0.700–0.736)	0.747 (0.731–0.763)	0.744 (0.726–0.762)
	No	76 458 (65.1)	0.83	0.797 (0.781–0.813)	0.817 (0.803–0.831)	0.814 (0.798–0.830)
eGFR, mL/min per 1.73 $m^{2}$	≥60	108 543 (92.4)	0.95	0.779 (0.767–0.791)	0.799 (0.787–0.811)	0.795 (0.783–0.807)
	30–60	6486 (5.5)	3.42	0.643 (0.610–0.676)	0.688 (0.655–0.721)	0.676 (0.643–0.709)
	<30	2494 (2.1)	5.65	0.645 (0.602–0.688)	0.648 (0.605–0.691)	0.637 (0.594–0.680)
Heart failure	Yes	1456 (1.2)	8.45	0.572 (0.527–0.617)	0.688 (0.641–0.735)	0.684 (0.637–0.731)
	No	116 067 (98.8)	1.10	0.781 (0.769–0.793)	0.79 (0.778–0.802)	0.786 (0.774–0.798)
Cardiovascular disease	Yes	18 959 (16.1)	2.37	0.686 (0.662–0.710)	0.722 (0.700–0.744)	0.719 (0.697–0.741)
	No	98 564 (83.9)	0.96	0.787 (0.773–0.801)	0.804 (0.792–0.816)	0.801 (0.789–0.813)
Stroke	Yes	6240 (5.3)	2.93	0.67 (0.635–0.705)	0.706 (0.671–0.741)	0.703 (0.668–0.738)
	No	111 283 (94.7)	1.09	0.779 (0.767–0.791)	0.799 (0.787–0.811)	0.795 (0.783–0.807)

AF indicates atrial fibrillation; and eGFR, estimated glomerular filtration rate.

**Figure 4:**
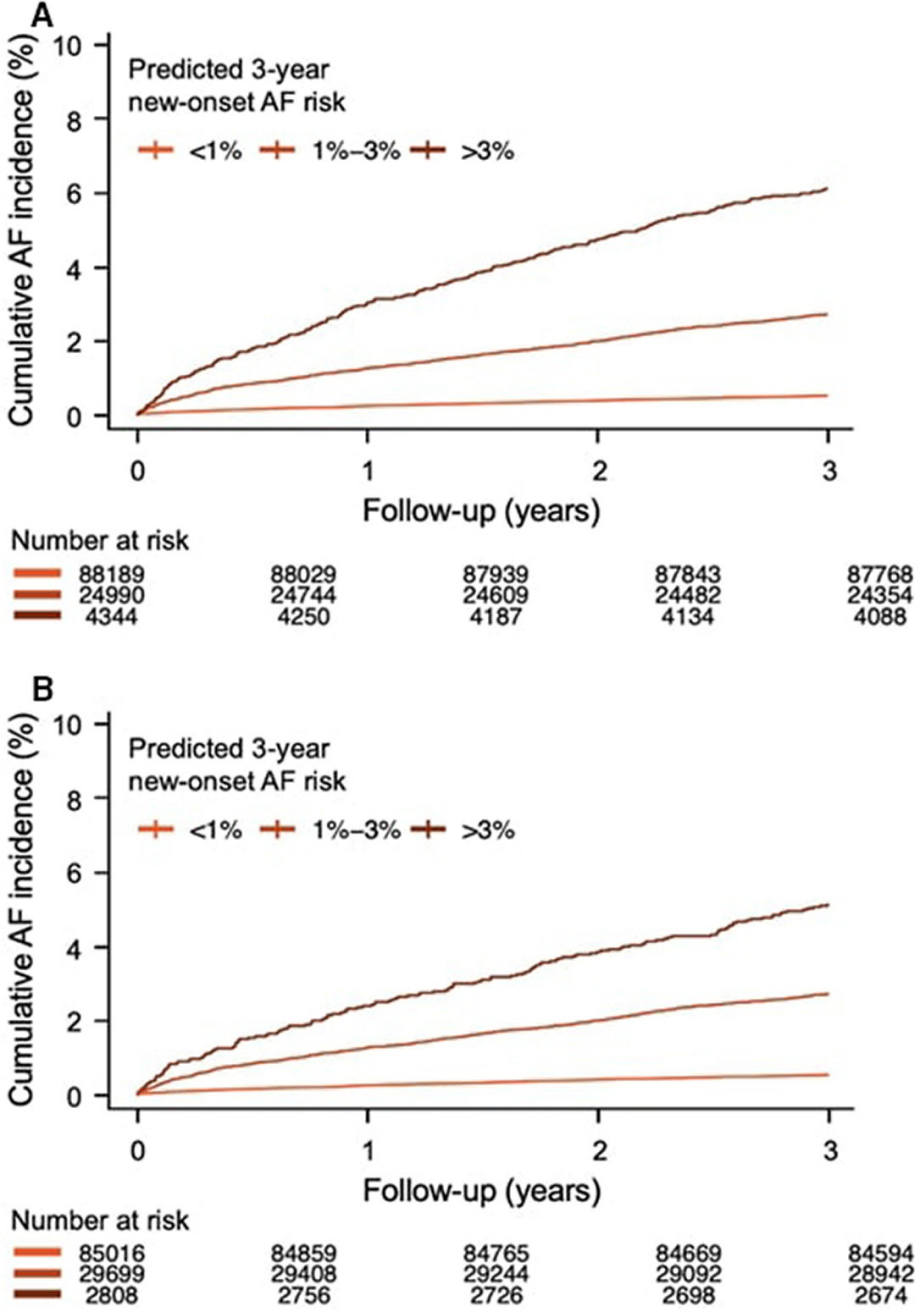
Cumulative incidence of atrial fibrillation (AF), stratified by the predicted risk based on the proposed model in the total validation cohort population. (**A**) Full model with ECG diagnosis (model 3). (**B**) Simplified model with ECG diagnosis (model 2).

Next, we divided the patients into 3 groups according to the estimated AF risk derived from the proposed models (low‐risk group, <1%; intermediate‐risk group, 1%–3%; and high‐risk group, >3%). Kaplan‐Meier curves showed time‐dependent linear and increased AF incidence across the 3 groups in both model 2 and model 3 (Figure 4). In model 3, the cumulative 3‐year AF incidence rate was 0.51%, 2.72%, and 6.12% in the low‐risk, intermediate‐risk, and high‐risk groups, respectively. In model 2, the cumulative 3‐year AF incidence rate was 0.52%, 2.71%, and 5.13% in the low‐risk, intermediate‐risk, and high‐risk tertiles, respectively. In model 3, compared with the low‐risk group, the intermediate‐risk group had a 5.39‐fold increased hazard for AF incidence (95% CI, 4.78–6.07; *P*<0.001) and the high‐risk group had a 12.36‐fold increased hazard for AF incidence (95% CI, 10.62–14.38; *P*<0.001). In model 2, compared with the low‐risk group, the intermediate‐risk group had a 5.22‐fold increased hazard for AF incidence (95% CI, 4.65–5.87; *P*<0.001), and the high‐risk group had a 10.01‐fold increased hazard for AF incidence (95% CI, 8.29–12.07; *P*<0.001).

### Comparison of the Proposed Models With the Previously Published Models (EHR‐AF, CHARGE‐AF, and C2HEST)

**Figure 5:**
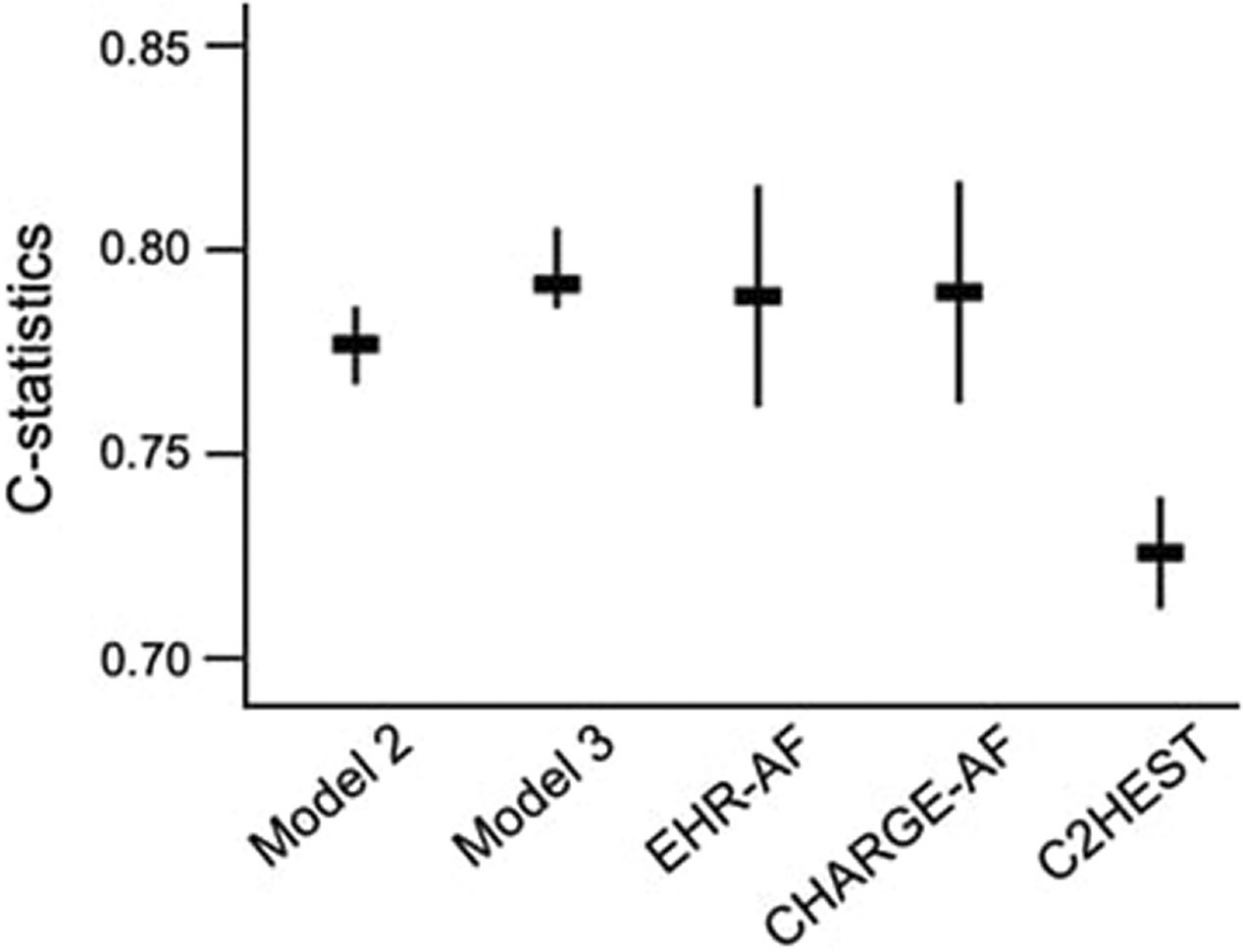
Comparison of C‐statistics for the models of the present study (model 2 and model 3), electronic health record–atrial fibrillation (EHR‐AF), Cohorts for Heart and Aging Research in Genomic Epidemiology Model for Atrial Fibrillation (CHARGE‐AF), and coronary artery disease or chronic obstructive pulmonary disease, hypertension, elderly, systolic heart failure, and thyroid disease (C2HEST) in the total validation cohort population. C‐statistics of EHR‐AF, CHARGE‐AF, and C2HEST were calculated from the total validation cohort. The original coefficients of the selected variables in EHR‐AF, CHARGE‐AF, and C2HEST were applied.

The AF predictive power of the models in the present study and the previously developed models, such as EHR‐AF, CHARGE‐AF, and C2HEST, was compared (Figure 5). We adopted all the predictors in the previously developed model. The C‐statistic was highest in model 3 (C‐statistic, 0.796 [95% CI, 0.785–0.806]) and lowest in C2HEST (C‐statistic, 0.725 [95% CI, 0.711–0.739]). The C‐statistic in model 2 (C‐statistic, 0.777 [95% CI, 0.766–0.788]) was slightly lower than those in the EHR‐AF (C‐statistic, 0.789 [95% CI, 0.762–0.816]) and CHARGE‐AF (C‐statistic, 0.790 [95% CI, 0.763–0.817]) models, and higher than that in the C2HEST model. These results suggest that model 2 can effectively predict new‐onset AF occurrence by adopting only age, sex, and computerized ECG diagnosis.

## Discussion

In the present study, we developed clinically relevant models for 3‐year new‐onset AF prediction. Because AF diagnosis was confirmed by ECG, we initially aimed to predict AF using only ECG data (model 1). With a systematic statistical approach, the following 5 ECG diagnoses were chosen: atrioventricular block, fusion beats, sinus arrhythmia (marked), supraventricular complex, and wide QRS complex. By adding minimal demographic variables (age and sex) to 5 ECG diagnoses, model 2 (simplified model with ECG diagnosis) showed improved C‐statistics (0.785 in the derivation cohort, and 0.777 in the total validation cohort). Its prediction efficacy was comparable to that of the full model with ECG diagnosis (model 3) or without ECG diagnosis (model 4), which included 6 clinical variables (age, male sex, CKD, heart failure, valvular heart disease, and previous stroke).

More accurate new‐onset AF prediction is possible with more data and clinical information. Nevertheless, a simplification of the model would be helpful, considering that there are practical limitations in obtaining a wide variety of accurate clinical information in clinical practice. Recently, attempts have been made to apply this clinical information more easily using EHRs. For this, the format and data of the EHRs should be standardized and managed consistently. In this study, an ECG diagnosis‐focused model was presented by using the automated readout of an ECG machine, which was transformed into a standardized ECG diagnosis.

Previously, AF prediction models using EHR data have been developed. For example, Aronson et al introduced a community‐based health care database‐driven AF prediction model using 10 variables (C‐statistic, 0.749).^17^ In addition, Hulme et al developed an AF prediction model using 16 EHR‐driven variables (C‐statistic, 0.777),^8^ whereas Tiwari et al developed an AF prediction model using 200 variables in the OMOP‐CDM database, which was transformed from EHR (AUC, 0.800).^18^ More recently, Grout et al developed an AF prediction model using 10 variables from the EHR (C‐statistic, 0.81).^19^ All the prior EHR‐driven AF prediction models used clinical variables that mainly originated from diagnosis codes or simple body measurements. The models of the present study adopted the detailed information of 130 ECG diagnoses that were generated and transformed from the computerized ECG machine.

In the past, ECG was usually performed in hospitals, but the recent development and wide use of personal ECG devices markedly improved the accessibility to ECG information. The previous CHARGE‐AF model showed the potential of ECG information to improve AF prediction in its augmented model, which added only 2 ECG‐related data (left ventricular hypertrophy and PR interval).^13^ The present study proposes 5 ECG diagnoses carefully extracted from 130 ECG diagnoses as important predictors of new‐onset AF incidence.

The AF development process might be summarized as abnormal atrial substrates and abnormal triggers through diverse pathways.^20^ Before clinical AF diagnosis, electrical abnormalities associated with these processes might be found at various distant points, such as heart failure and valvular heart diseases. It can be inferred that the ECG findings predicting AF imply electrical or structural cardiac abnormalities that contribute to AF development. The proposed ECG findings for AF prediction in the present study included atrial abnormal *triggers* (fusion beats, supraventricular premature complex, and wide QRS rhythm) as well as abnormal *substrate* (marked sinus arrhythmia and atrioventricular block).

In the present study, a model using ECG diagnosis (model 2) along with minimal demographic information of age and sex (men) showed an AF prediction potential comparable to those of the prior predictive models using >10 clinical information variables that can be obtained through a physician's questionnaire. Thus, model 2 using ECG information could have a crucial advantage in real‐world applications. ECG information can be easily obtained without extra cost or effort through a computerized interpretation system. Although many clinicians agree that AF risk prediction is important, they are often skeptical of gathering information for prediction. This is probably because they might not have found the results of the previous conventional AF prediction models, which adopted clinical variables, to be significantly different from what they already knew empirically. Finally, the ECG‐driven AF prediction model (model 2) can be applied to health checkups for the general population. Moreover, its performance was better in subjects without AF risk factors than in those with AF risk factors.

## Limitations

There are several limitations to the present study. First, in our data, ≈87% of the eligible candidates had missing values, so we excluded them. These excluded candidates were relatively younger and healthier than the candidates without missing values (Table S2). There is a potential risk of selection bias. Second, this was a retrospective study. Patients with AF at the time of study enrollment were not screened by the Holter test but only by 12‐lead ECG. Thus, patients with subclinical AF were not completely excluded at the time of enrollment. However, AF occurrence showed a linear relationship (Figure 4), suggesting that subclinical AF was sufficiently excluded. Third, only 12‐lead ECG data were used in the present study. Holter test results, which contained more ECG data, were not used. If Holter monitor or wearable ECG devices were used for new‐onset AF modeling, an algorithm with better predictive power could be developed. Finally, the present study adopted an ECG diagnosis transformed from a computerized ECG interpretation system. It did not use the original raw ECG wave data; therefore, if it was modeled using the original ECG waveform data, the results may have been different or even improved.

## Conclusions

The present study developed predictive models for 3‐year AF occurrence using EHR data, including ECG diagnosis. Although the model that included many variables had the best predictive power, the model that simply added age and sex to the ECG diagnosis also showed a comparable predictive power with broad applicability for incident AF.

## Sources of Funding

This research was supported by a grant from the Korea Health Technology R&D Project through the Korea Health Industry Development Institute and the Chung‐Ang University Research Grants in 2020. This study was funded by the Ministry of Health and Welfare, Republic of Korea (grant: HI19C0360).

## Disclosures

Dr Gregory Y. H. Lip is a consultant and speaker for BMS/Pfizer, Boehringer Ingelheim and Daiichi‐Sankyo (no fees are received personally). Dr Hyung Joon Joo reports research support and lecture fees from Hanmi, Daewoong, Dio, HK and Inno.N.

## Supporting information

Table S1–S2
