# Supplementary material for: Development and Validation of 3‐Year Atrial Fibrillation Prediction Models Using Electronic Health Record With or Without Standardized Electrocardiogram Diagnosis and a Performance Comparison Among Models
Source: J Am Heart Assoc. 2022 Jun 14;11(12):e024045. doi: 10.1161/JAHA.121.024045 (PMC9238645; doi:10.1161/JAHA.121.024045)
Supplement: Supplementary file 1 — Table S1–S2 [file JAH3-11-e024045-s001.pdf]

SUPPLEMENTAL MATERIAL

Table S1. Univariate Cox regression analysis of clinical risk factors and ECG diagnosis for 3-year new-onset AF prediction in the derivation cohort.

Variable	HR (95 % CI)	*P* Value
Age, years	1.06 (1.05–1.07)	<.001
Male	1.44 (1.13–1.83)	0.003
Current smoking	1.14 (0.84–1.55)	0.405
Alcohol drinking	0.90 (0.67–1.22)	0.505
Systolic BP, mmHg	1.02 (1.02–1.03)	<.001
Diastolic BP, mmHg	1.02 (1.01–1.03)	<.001
Hypertension	3.06 (2.41–3.89)	<.001
Diabetes Mellitus	2.32 (1.83–2.94)	<.001
Insulin	1.82 (1.08–3.07)	0.024
Dyslipidemia	2.51 (1.97–3.18)	<.001
Thyroid disease	1.07 (0.48–2.40)	0.874
COPD	3.78 (0.53–26.94)	0.184
Heart failure	10.11 (6.41–15.95)	<.001
MV stenosis	13.46 (3.35–53.99)	<.001
VHD except MV stenosis	8.19 (3.38–19.83)	<.001
Coronary artery disease	2.81 (2.02–3.91)	<.001

Stroke	4.09 (2.97–5.63)	<.001
Peripheral arterial disease	1.03 (0.14–7.31)	0.980
Creatinine, mg/dL	1.20 (1.13–1.26)	<.001
ECG		
Aberrant conduction complex	5.65 (1.40–22.69)	0.015
Atrial premature complex	7.43 (4.17–13.25)	<.001
AV block	4.45 (2.76–7.17)	<.001
AV block (1st degree)	3.89 (2.35–6.45)	<.001
AV block (2:1)	14.95 (2.10–106.55)	0.007
AV block (2nd degree)	12.91 (1.81–91.95)	0.011
AV dissociation	44.29 (6.23–315.00)	<.001
Early repolarization	0.41 (0.06–2.94)	0.377
Fusion beat	22.52 (9.30–54.53)	<.001
Intra-ventricular conduction defect	6.62 (1.65–26.61)	0.008
LAFB	3.66 (1.36–9.82)	0.010
LBBB	6.98 (2.24–21.76)	0.001
Left axis deviation	2.08 (1.10–3.90)	0.023
LVH	2.21 (1.31–3.72)	0.003

Myocardial infarction	3.99 (0.99–16.03)	0.051
Myocardial infarction (anterior)	3.99 (1.49–10.71)	0.006
Myocardial infarction (antero-lateral)	3.56 (0.50–25.33)	0.205
Myocardial infarction (antero-septal)	1.41 (0.20–10.01)	0.733
Myocardial infarction (inferior)	0.68 (0.22–2.12)	0.507
Myocardial infarction (lateral)	2.51 (0.63–10.10)	0.194
Myocardial infarction (posterior)	1.82 (0.26–12.99)	0.549
Myocardial infarction (septal)	2.24 (0.72–6.96)	0.165
Myocardial ischemia (anterior)	2.49 (1.23–5.04)	0.011
Myocardial ischemia (inferior)	2.00 (0.64–6.22)	0.234
Myocardial ischemia (lateral)	1.98 (0.98–4.01)	0.056
Normal sinus rhythm	0.48 (0.36–0.63)	<.001
P wave (left atrial enlargement)	3.00 (1.12–8.04)	0.029
PR interval (short)	0.96 (0.13–6.80)	0.964
QT interval (prolonged)	2.67 (1.59–4.49)	<.001
R wave (abnormal)	9.03 (6.89–348.74)	<.001
RBBB	1.17 (0.43–3.13)	0.761
RBBB (incomplete)	2.98 (1.11–7.99)	0.030

Right axis deviation	0.67 (0.21–2.08)	0.487
rSr pattern in V1 and V2	2.41 (0.34–17.16)	0.380
Sinus arrhythmia	1.88 (0.97–3.66)	0.062
Sinus arrhythmia (marked)	10.28 (4.86–21.78)	<.001
Sinus rhythm	3.55 (2.54–4.97)	<.001
Sinus rhythm (bradycardia)	1.14 (0.74–1.76)	0.554
Sinus rhythm (tachycardia)	1.62 (0.86–3.04)	0.135
ST segment change	2.76 (0.69–11.08)	0.153
ST segment depression	5.84 (0.82–41.54)	0.078
ST segment elevation	1.90 (0.61–5.94)	0.268
ST segment elevation (lateral)	32.21 (4.52–229.51)	0.001
ST-T abnormality (non-specific)	2.18 (1.08–4.40)	0.030
Supraventricular premature complex	17.21 (8.52–34.76)	<.001
T wave (abnormal)	1.63 (1.04–2.54)	0.032
Ventricular premature complex	5.48 (3.07–9.77)	<.001
Voltage (decreased)	2.76 (1.03–7.42)	0.044
Wide QRS complex	11.98 (6.16–23.27)	<.001

ECG diagnoses for which statistics were not calculated in univariate analysis were excluded from the table. BP, blood pressure; COPD, chronic obstructive pulmonary disease; MV, mitral valve; VHD, valvular heart disease; LAFB, left anterior fascicular block; LBBB, left bundle branch block; LVH, left ventricular hypertrophy; RBBB, right bundle branch block; HR, hazard ratio; CI, confidence interval.

Table S2. Baseline comparison between missing value population and non-missing value population.

	**Non-missing (n = 51,167)**	**Missing (n = 342,796)**	***P* Value**
Clinical characteristics			
Age, years	50.56 ± 21.77	44.23 ± 22.04	<.001
Male	25,910 (50.64)	159,397 (46.5)	<.001
Current smoker	8,284 (16.19)	980 (0.29)	<.001
Alcohol drinking	11,124 (21.74)	1,353 (0.39)	<.001
Hypertension	12,859 (25.13)	22,118 (6.45)	<.001
Diabetes mellitus	17,489 (34.18)	22,707 (6.62)	<.001
Dyslipidemia	13,324 (26.04)	23,390 (6.82)	<.001
Chronic kidney disease	942 (1.84)	2,543 (0.74)	<.001
Thyroid disease	1,035 (2.02)	4,387 (1.28)	<.001
COPD	48 (0.09)	116 (0.03)	<.001
Heart failure	417 (0.81)	636 (0.19)	<.001
VHD			<.001
MV stenosis	22 (0.04)	79 (0.02)	
Other VHD	128 (0.25)	341 (0.1)	
Coronary artery disease	3,157 (6.17)	7,665 (2.24)	<.001
Stroke	2,423 (4.74)	3,239 (0.94)	<.001
Peripheral arterial disease	194 (0.38)	1,063 (0.31)	0.010
ECG diagnosis (top 20)			
Normal sinus rhythm	19,537 (38.18)	22,517 (6.57)	<.001
Sinus rhythm (bradycardia)	3,676 (7.18)	5,512 (1.61)	<.001

T wave (abnormal)	2,478 (4.84)	1,970 (0.57)	<.001
Sinus rhythm	2,430 (4.75)	2,841 (0.83)	<.001
LVH	1,288 (2.52)	1,418 (0.41)	<.001
Sinus rhythm (tachycardia)	1,174 (2.29)	594 (0.17)	<.001
QT interval (prolonged)	1,048 (2.05)	609 (0.18)	<.001
Sinus arrhythmia	944 (1.84)	1,292 (0.38)	<.001
Left axis deviation	901 (1.76)	981 (0.29)	<.001
Right axis deviation	869 (1.7)	1,109 (0.32)	<.001
AV block (1st degree)	838 (1.64)	1,024 (0.3)	<.001
AV block	825 (1.61)	1,049 (0.31)	<.001
Myocardial infarction (inferior)	799 (1.56)	800 (0.23)	<.001
Myocardial ischemia (lateral)	762 (1.49)	555 (0.16)	<.001
ST-T abnormality (non-specific)	652 (1.27)	578 (0.17)	<.001
RBBB	636 (1.24)	722 (0.21)	<.001
Myocardial ischemia (anterior)	607 (1.19)	395 (0.12)	<.001
Early repolarization	477 (0.93)	684 (0.2)	<.001
Ventricular premature complex	442 (0.86)	816 (0.24)	<.001
ST segment elevation	334 (0.65)	380 (0.11)	<.001

Values are presented as n (%) or mean ± standard deviation. AV, atrioventricular; BP, blood pressure; COPD, chronic obstructive pulmonary disease; LVH, left ventricular hypertrophy; MV, mitral valve; VHD, valvular heart disease; RBBB, right bundle branch block; ECG, electrocardiogram
